# Supplementary material for: Differential Membrane Lipid Profiles and Vibrational Spectra of Three Edaphic Algae and One Cyanobacterium
Source: Int J Mol Sci. 2021 Oct 19;22(20):11277. doi: 10.3390/ijms222011277 (PMC8538821; doi:10.3390/ijms222011277)
Supplement: Supplementary file 1 [file ijms-22-11277-s001.zip › ijms-1410479-supplementary.pdf]

Differential membrane lipid profiles and vibrational spectra of three edaphic algae and one cyanobacterium

Olimpio Montero[1,*], Marta Velasco[1], Jorge Miñón[2], Evan A.N. Marks[3], Aurelio Sanz-Arranz[4], and Carlos Rad[2]

[1] Institute of Biology and Molecular Genetics (IBGM), Spanish Council for Scientific Research (CSIC), Sanz y Forés Str. 3 47003-Valladolid, Spain. E-mails: olimpio.montero@dicyl.csic.es (O.M.), Orcid: 0000-0002-0241-8756; m.velasco@dicyl.csic.es (M.V.)
[2] Composting Research Group UBUCOMP, Faculty of Sciences, University of Burgos, 09001 Burgos, Spain. E-mails: jorge.minon.martinez@gmail.com (J.M.); crad@ubu.es (C.R.)
[3] BETA Technological Center, University of Vic–University of Central Catalonia, Edifici Can Baumann, Crta. de Roda 70, 08500 Vic, Catalonia, Spain. evan.marks@uvic.cat. Orcid: 0000-0002-2931-5976
[4] Fisica de la Materia Condensada Department, University of Valladolid, Valladolid, Spain.
* Correspondence: olimpio.montero@dicyl.csic.es; Tel.: +34-983-548-209

1. *Ribosomal small subunit sequencing*

For species identification, the 16S rDNA for cyanobacteria or the 18S rDNA for eukaryotic algae were sequenced and compared through nucleotide BLAST with available sequences at the U.S. National Center for Biotechnology Information (NCBI) (https://www.ncbi.nlm.nih.gov/). The primers used are depicted in Table S1.

Table S1. Primers used for the 16S and 18S rDNA extraction and PCR amplification. Data on similarity (%) and total sequence score (number of nucleotides compared) after the nucleotide BLAST NCBI database (https://www.ncbi.nlm.nih.gov/) are also depicted. C.P., combined primers (905F+1492R+1A+564R)[1]. Organisms: K, *Klebsormidium*; H, *Haslea*; M, *Microcoleus*.

Primer code	Primer sequence	Organism	Similarity (%)	TSS
C.P.	See footnote 1	*K. flaccidum*	99	951
C.P.	See footnote 1	*K. flaccidum*	100	894
C.P.	See footnotes 1,2	*Oocystis sp.*	98	900
C.P.	See footnotes 1,2	*Oocystis sp.*	98	878
C.P.	See footnote 1	*H. spicula*	99	933
C.P.	See footnote 1	*H. spicula*	99	898
P2F	5′-GGGGAATTTTCCGCAATGGG-3′	*M. vaginatus*	98	230
P1R	5'-CTCTGTGTGCCTAGGTATCC-3'	*M. vaginatus*	97	409
106F	5′-CGGACGGGTGAGTAACGCGTGA-3′	*A. salina*	99	473
738F	5′-ATACCCCWGTAGTCCTAGC-3′	*A. salina*	99	444
1492R	5′-GGTTACCTTGTTACGACTT-3′	*A. salina*	100	396

781R	5′-GACTACTGGGGTATCTAATCCCATT-3′	*A. salina*	100	514

[1] 905F = 5′-TGAAACTYAAAGGAATTG-3′, 1A = 5′-AACCTGGTTGATCCTGCCAGT-3′; 564R = 5′-GGCACCAGACTTGCCCTC-3′. [2] When these primers were used a high similarity was found with *Acutodesmus obliquus*, but total sequence score was low (<270), and further analysis was conducted to clarify this issue by using new primers (EC18SF, 5′-GGTTGATCCTGCCAGTAG-3′; EC18SR, 5'-TACGACTTCTCCTTCCTCTA-3').

2. *High performance liquid chromatography (HPLC-DAD) measurements*

Photosynthetic pigments in the methanolic extract were analyzed by high performance liquid chromatography with photodiode array detection (HPLC-DAD) using the same chromatographic method as in Montero et al. [25]. A FINNIGAN SURVEYOR PLUS chromatography system (Thermo Scientific) equipped with Quaternary LC Pump, Autosampler and PDA detector was used for HPLC-DAD measurements. Pigments were identified according to retention time and UV-Vis spectrum (350-700 nm). Regression curves were drawn for Chlorophyll *a* (Chla), Zeaxanthin (Z) and β-Carotene (bC) using commercial standards from SIGMA-ALDRICH (references are C5753 for Chla, 1733122 (USP) for Z, and 1065480 (USP) for bC). Chlorophylls, xanthophylls and carotenes were quantified using the regression parameters for chlorophyll *a*, zeaxanthin and β-carotene, respectively.

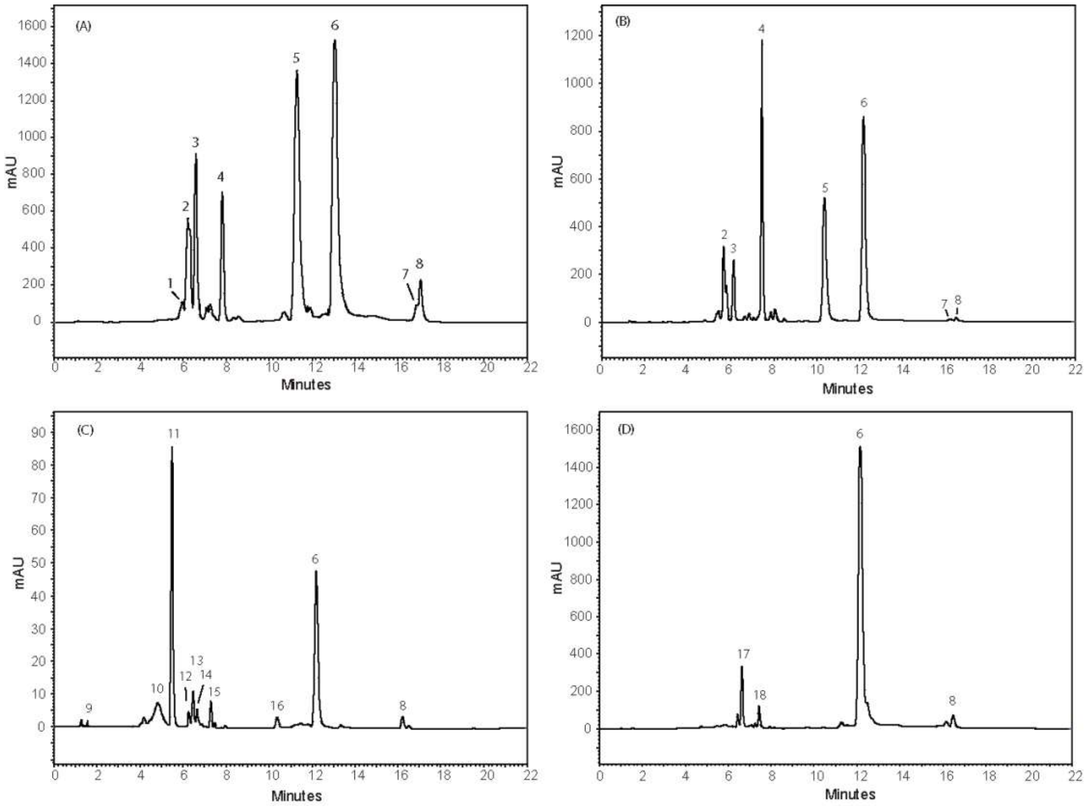

Supplementary Figure S1. Chromatograms obtained after HPLC-DAD analysis for the algae and the cyanobacterium studied here under the culture conditions used in this study. Each peak is shown at its own maximum absorption wavelength (Max-Plot). (A) *Klebsormidium flaccidum*; (B) *Oocystis* sp.; (C) *Haslea spicula*; (D) *Microcoleus vaginatus* Pigments (peaks): 1, unknown; 2, neoxanthin (+lutein epoxide in *Oocystis* sp.); 3, violaxanthin; 4, lutein (+zeaxanthin); 5, chlorophyll *b*; 6, chlorophyll *a*; 7, α-carotene; 8, β-carotene; 9, chlorophyll *c2*?; 10, fucoxanthinol; 11, fucoxanthin; 12, hexanoyl-fucoxanthin; 13, diadinoxanthin; 14, octanoyl-fucoxanthin; 15, diatoxanthin; 16, chlorophyll *c1*; 17, myxoxanthophyll; and 18, zeaxanthin.

3. *UPLC-QToF-MS measurements: Base peak (BPI) chromatograms*

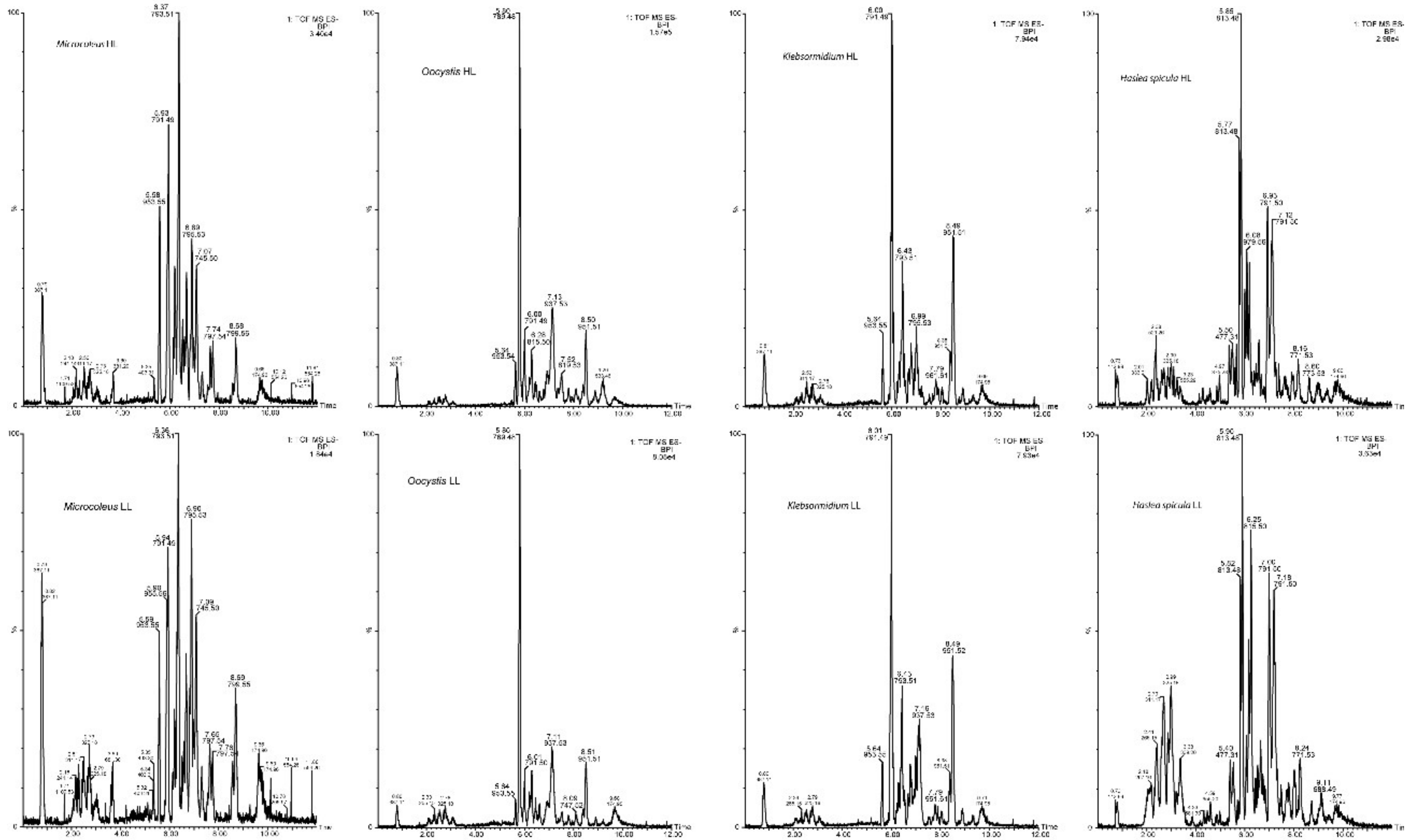

Supplementary Figure S2. Representative base peak chromatograms (BPI) obtained under negative ionization (ESI-) for the algal and cyanobacterial species under the culture conditions of the study after UPLC-ESI-QToF-MS analysis of a chloroform:methanol extract. LL, low light (15 µmol photons m^{-2} s^{-1}); HL, high light (45 µmol photons m^{-2} s^{-1}).

4. Raman and FTIR spectra

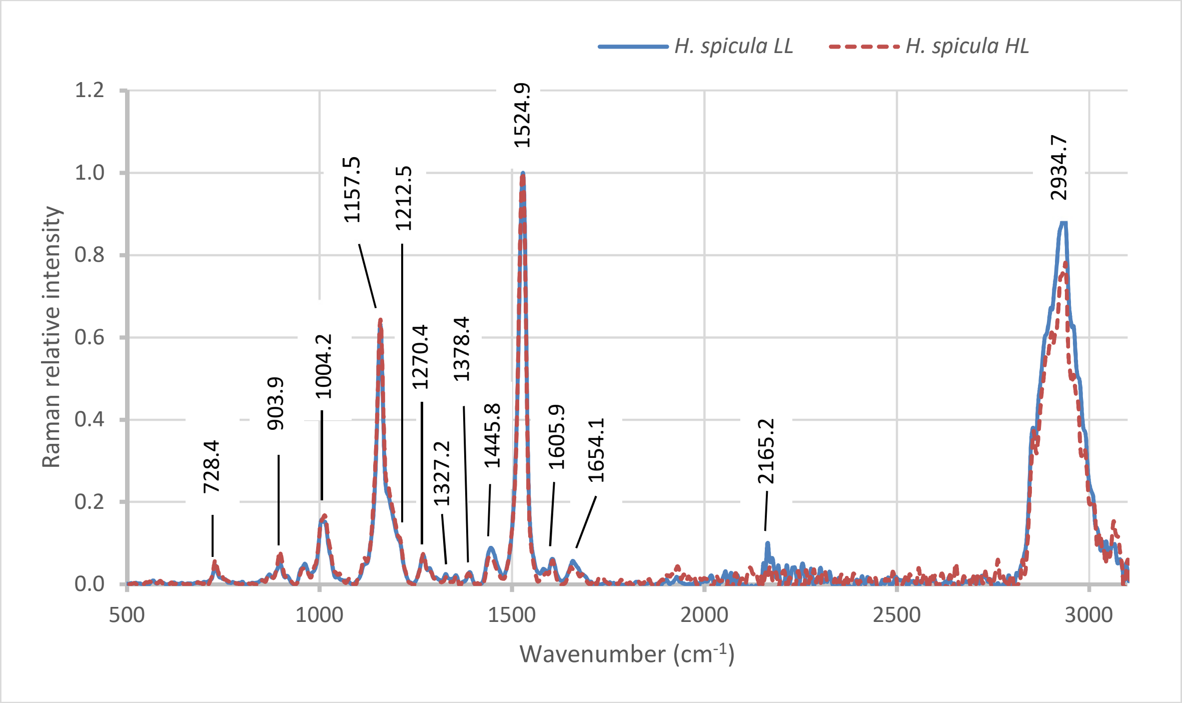

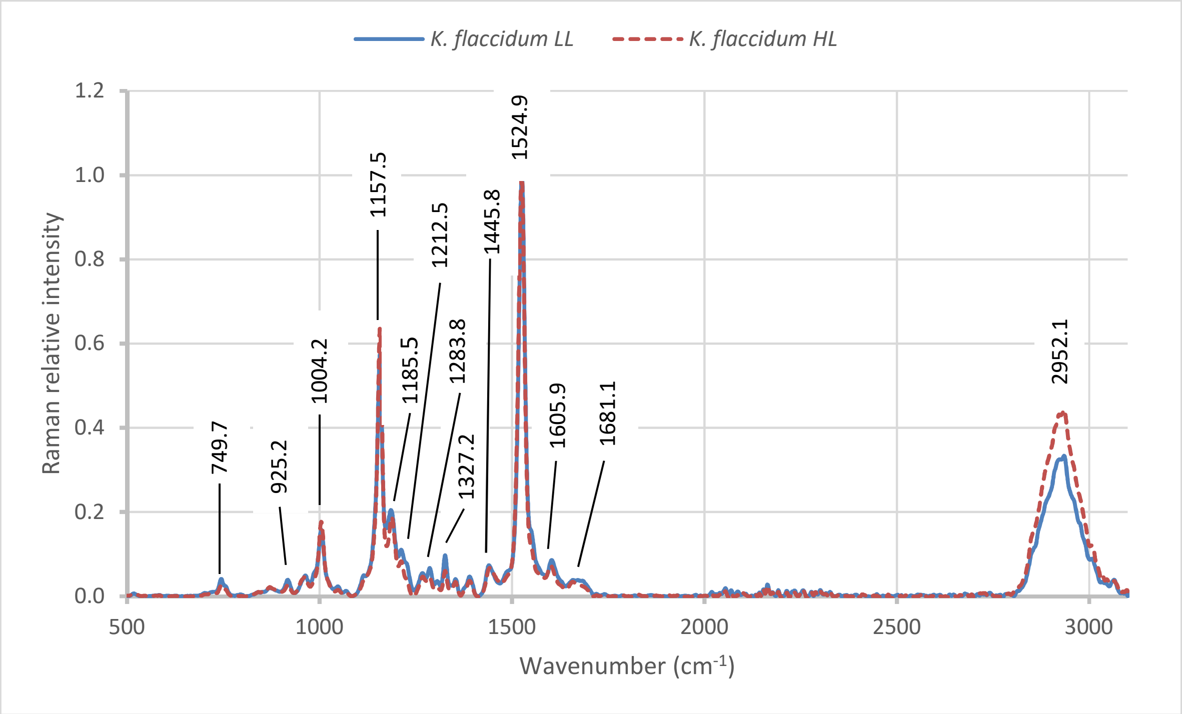

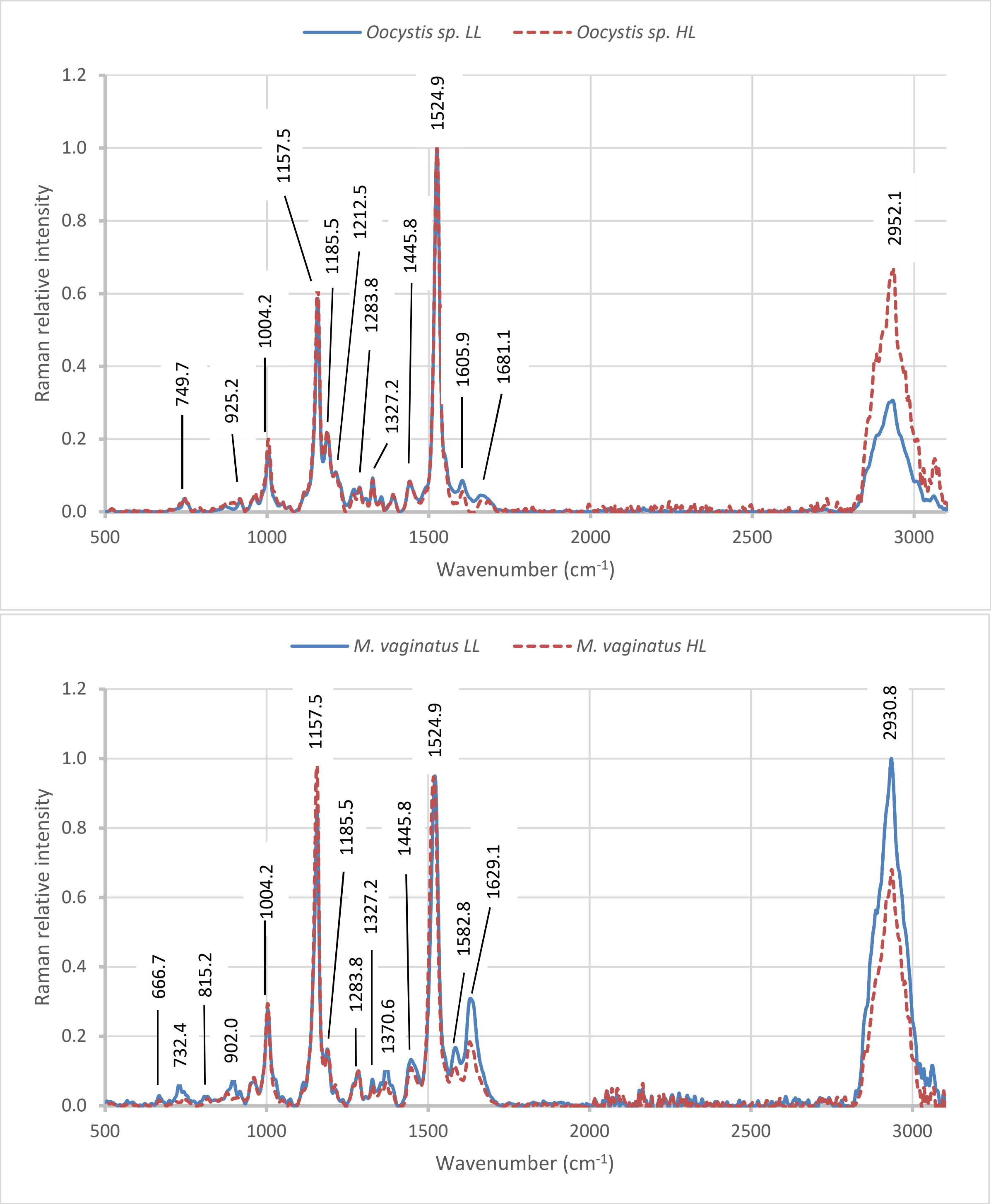

Supplementary Figure S3. Raman spectra of the different organisms used in this study under low light (blue line, 15 µmol photons m^{-2} s^{-1}) and high light (dashed red line, 45 µmol photons m^{-2} s^{-1}). Each spectrum was normalized to its absorption maximum. Abbreviations: K, *Klebsormidium*; H, *Haslea*; and M, *Microcoleus*.

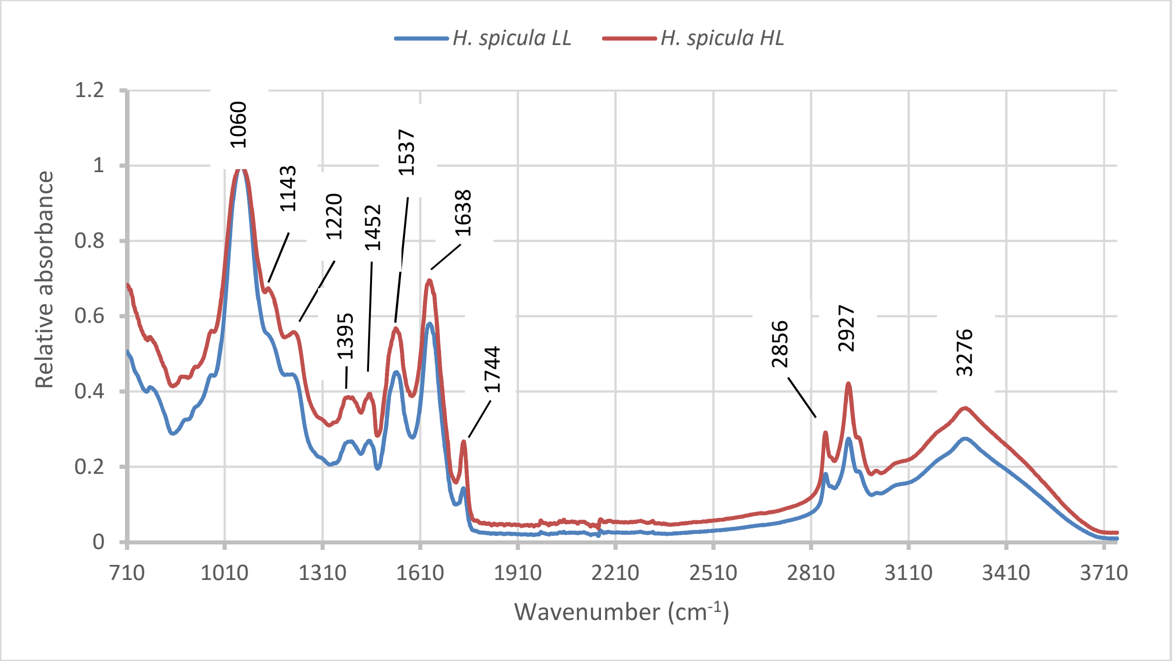
H. spicula LL
H. spicula HL
Relative absorbance
1.2
1
0.8
0.6
0.4
0.2
0
710
1010
1310
1610
1910
2210
2510
2810
3110
3410
3710
Wavenumber (cm-1)
1060
1143
1220
1395
1452
1537
1638
1744
2856
2927
3276

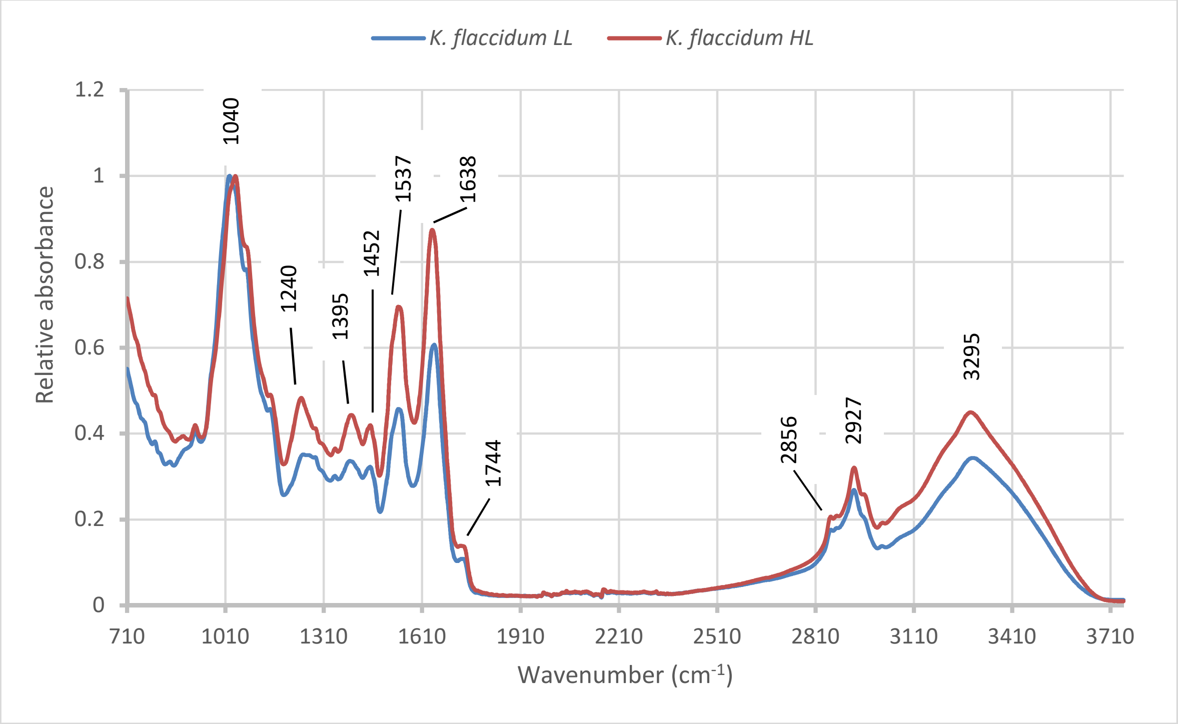
K. flaccidum LL
K. flaccidum HL
Relative absorbance
1.2
1
0.8
0.6
0.4
0.2
0
710
1010
1310
1610
1910
2210
2510
2810
3110
3410
3710
Wavenumber (cm-1)
1040
1240
1395
1452
1537
1638
1744
2856
2927
3295

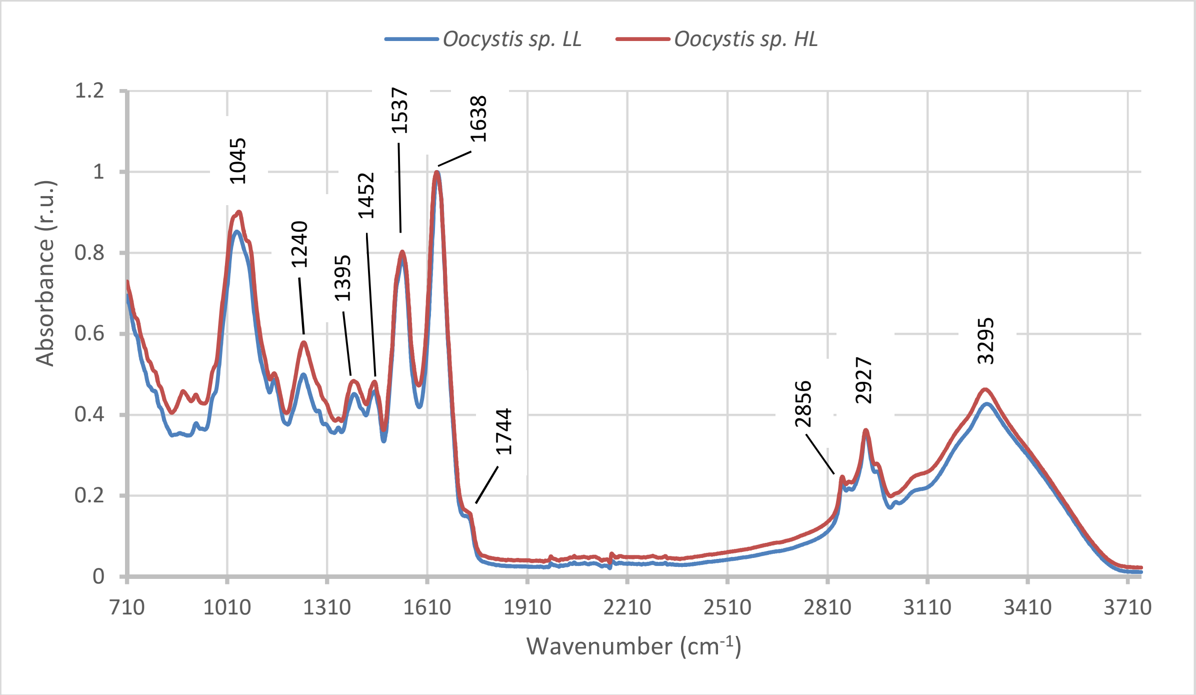

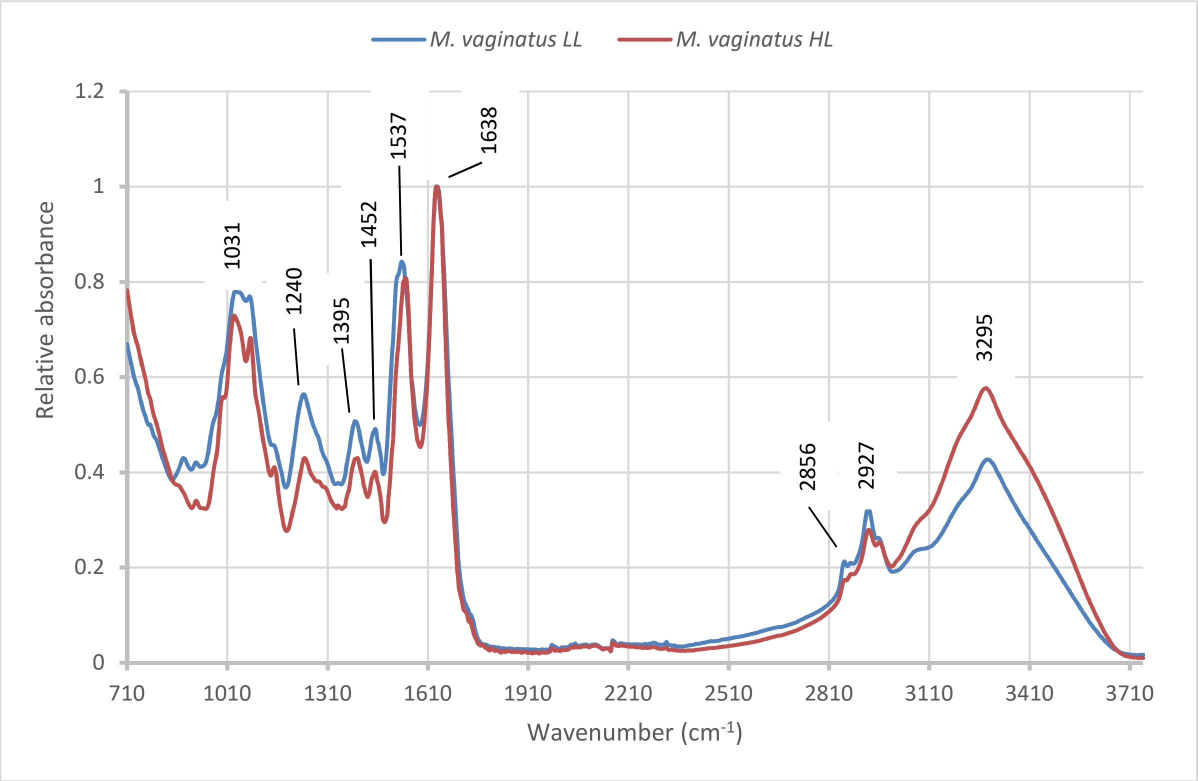

Supplementary Figure S4. FTIR spectra of the different organisms used in this study under low light (blue line, 15 µmol photons m^{-2} s^{-1}) and high light (red line, 45 µmol photons m^{-2} s^{-1}). Each spectrum was normalized to its absorption maximum. Abbreviations: K, *Klebsormidium*; H, *Haslea*; and M, *Microcoleus*.

ANNEX A

High quality figures 3 and 4 (only panels a, b, c and d)

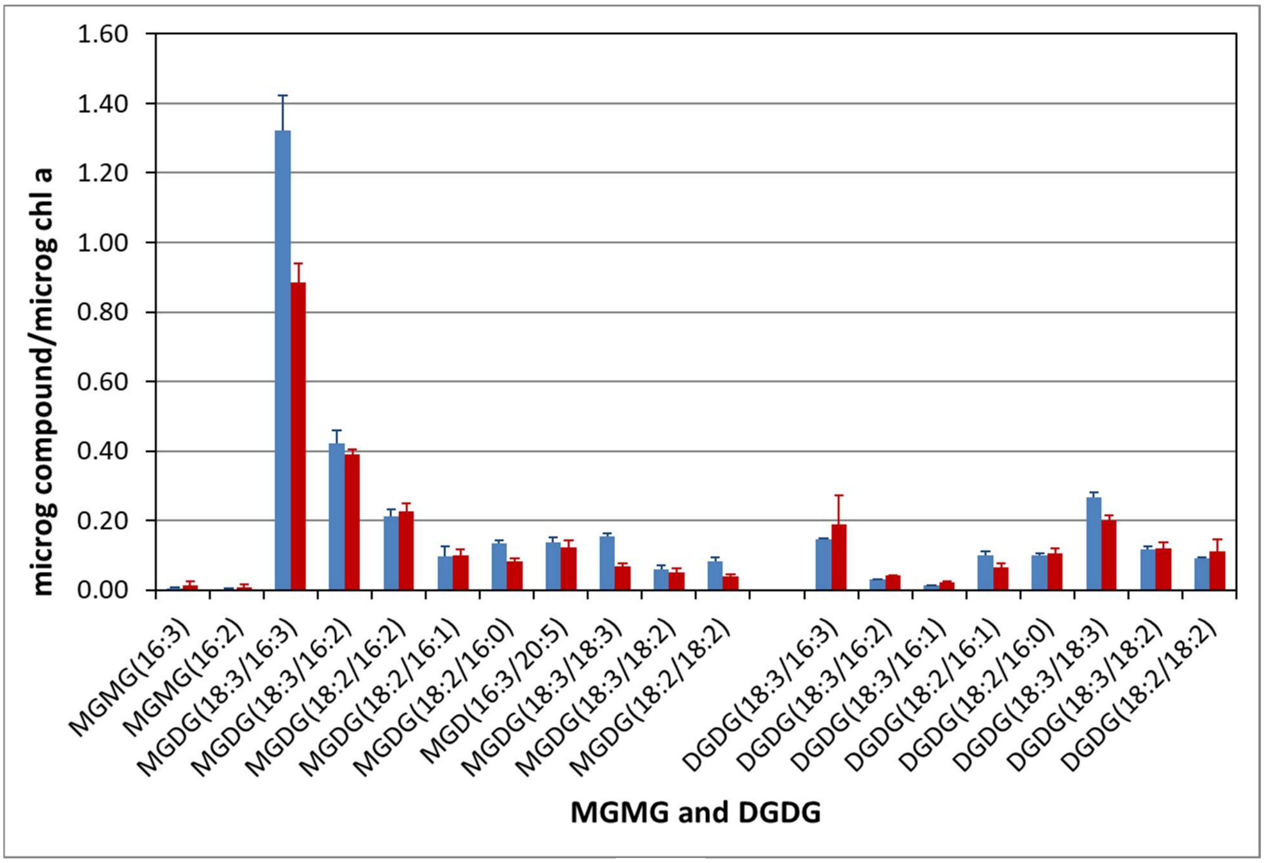

(a)

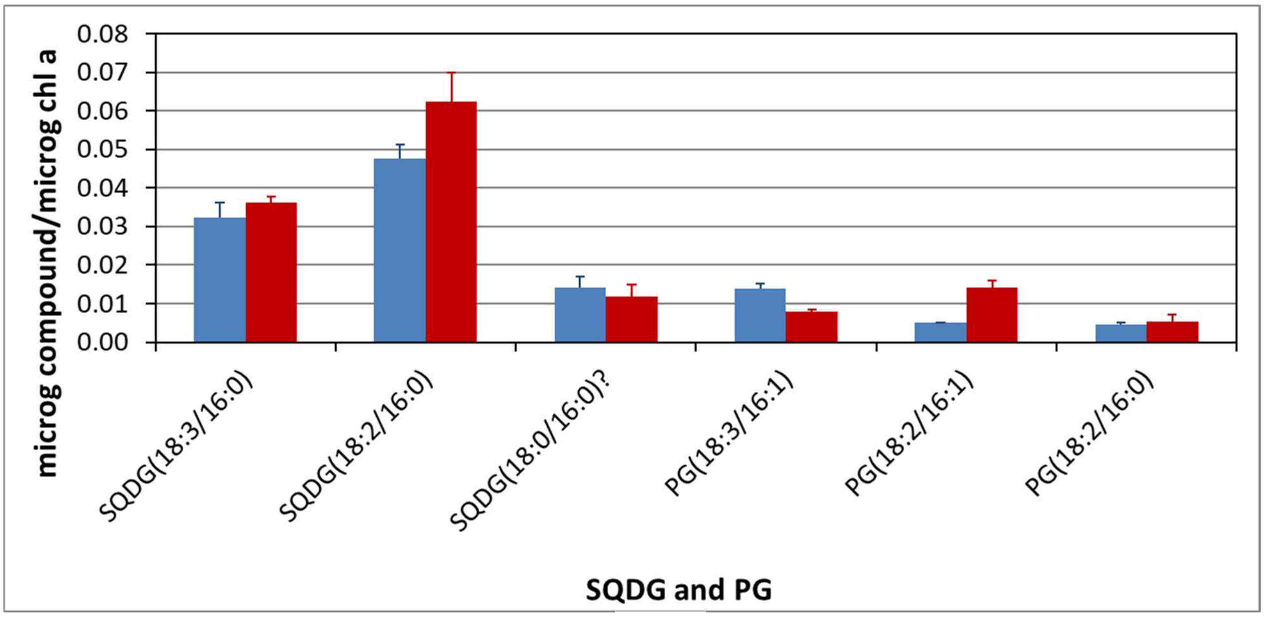

(c)

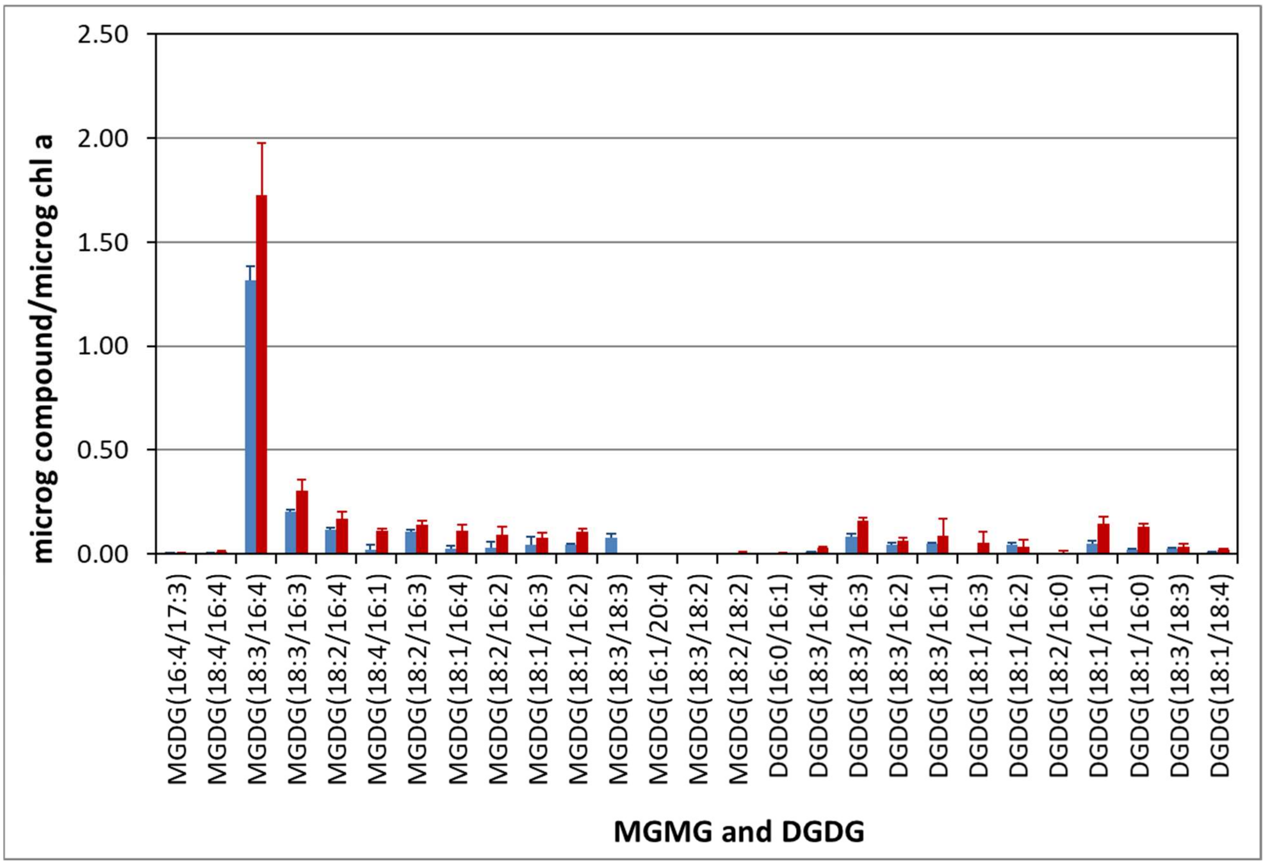

(b)

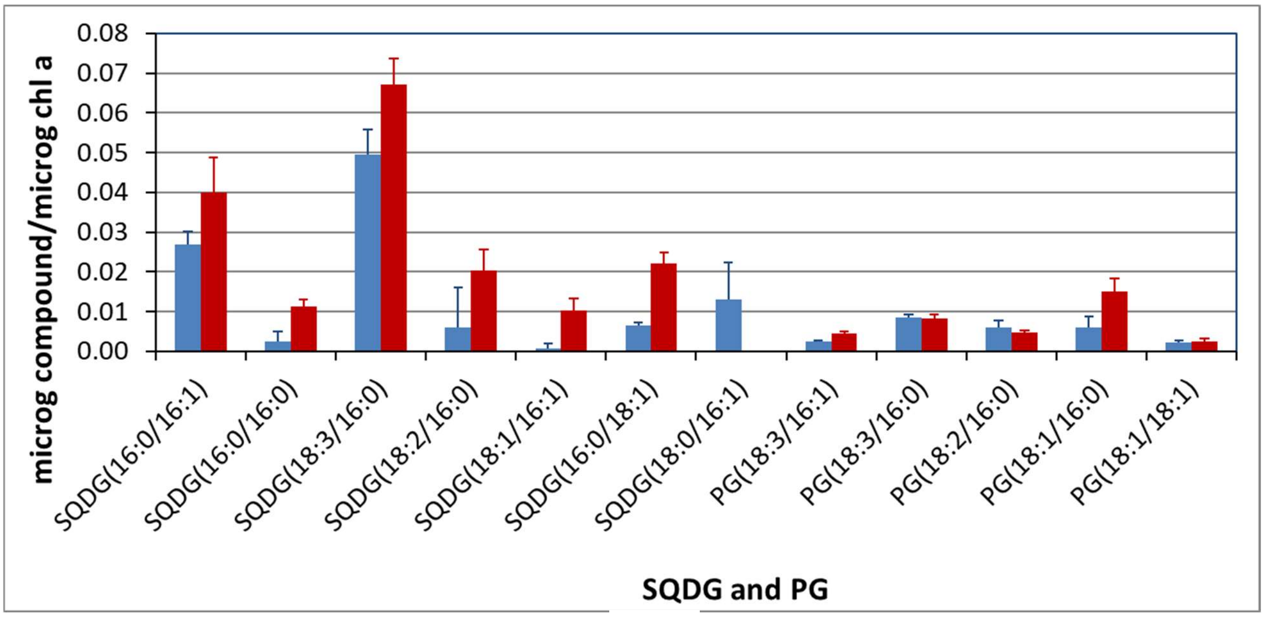

(d)

Figure 3. Content of the different glycerolipids detected in the extract of the algae *K. flaccidum* (panels a and c) and *Oocystis* sp. (panels b and d). MGMG: monogalactosylmonoacylglycerol; MGDG: monogalactosyldiacylglycerol; DGDG: digalactosyldiacylglycerol; SQDG: sulfoquinovosyldiacylglycerol; PG:

diacylglycerylphosphoglycerol. Values are the mean ± standard deviation of three independent cultures (n=3). Blue bars, low light; red bars, high light.

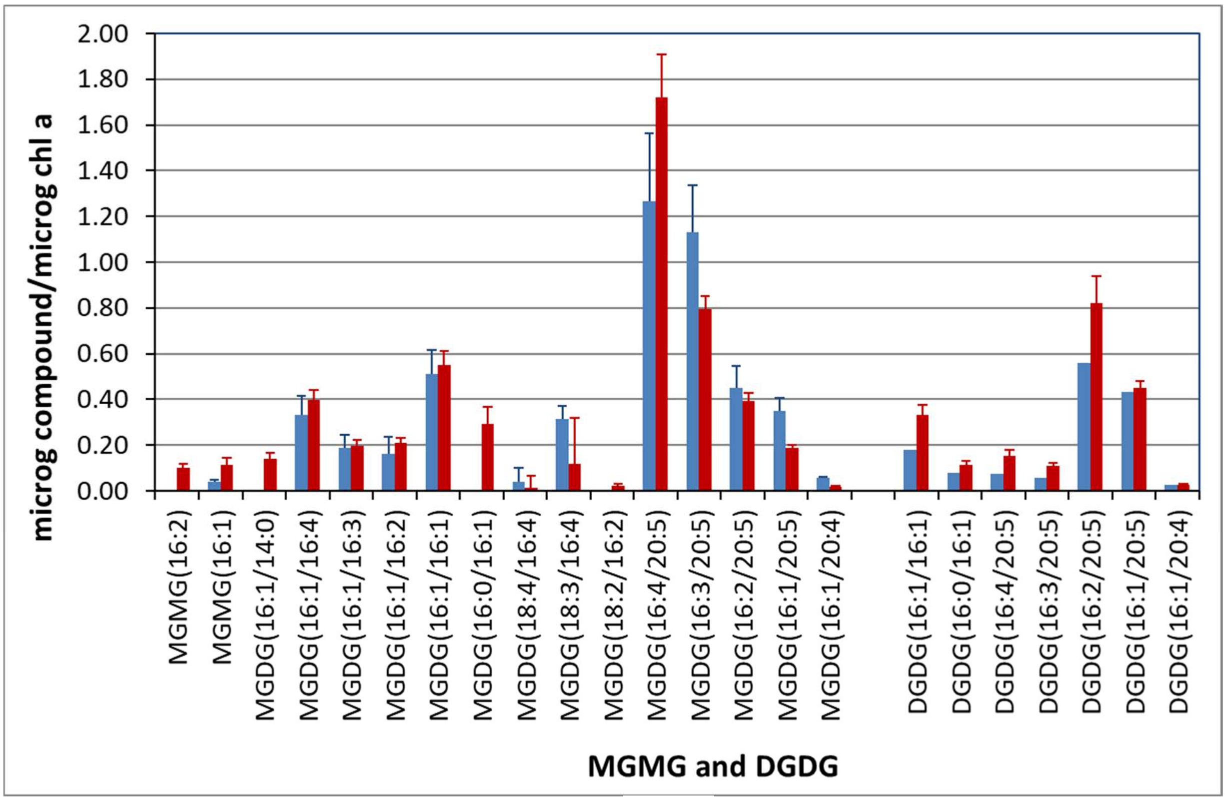

(a)

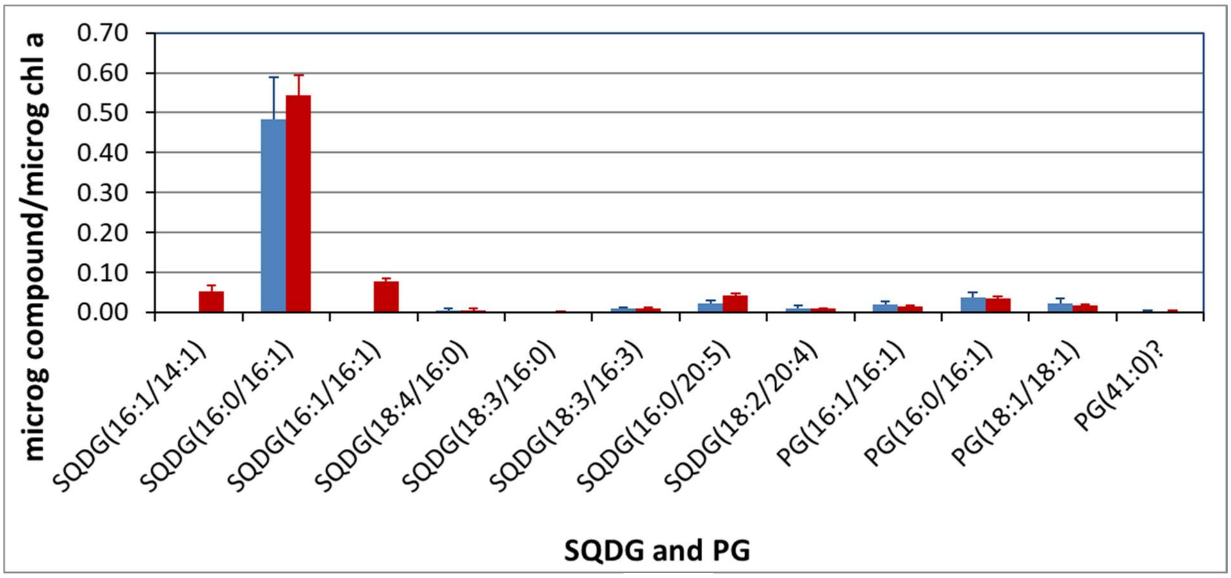

(c)

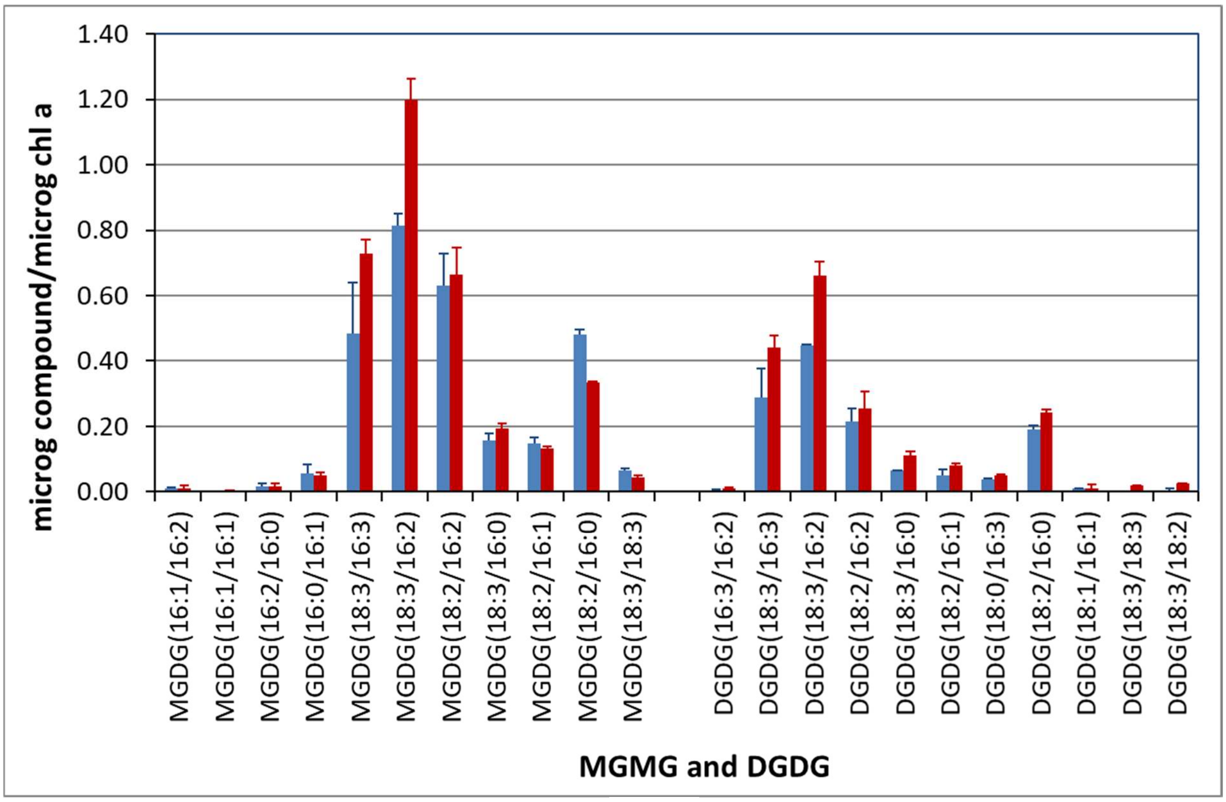

(b)

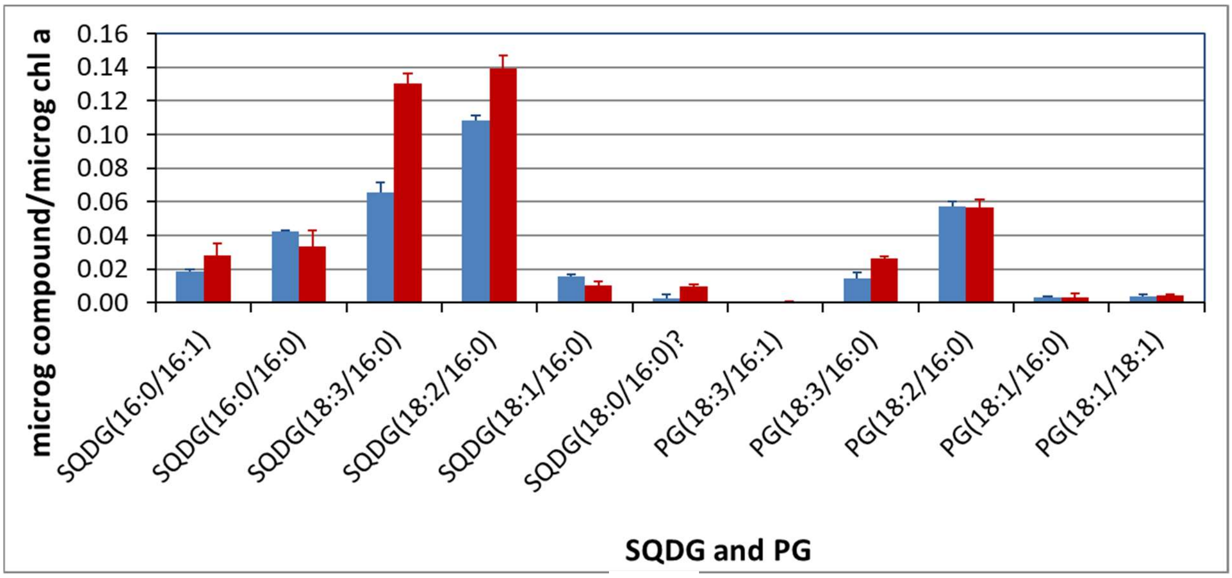

(d)

Figure 4. Content of the different glycerolipids detected in the extract of the algae *H. spicula* (panels A, B and C) and *M. vaginatus* (panels D and E). MGMG: monogalactosylmonoacylglycerol; MGDG: monogalactosyldiacylglycerol; DGDG: digalactosyldiacylglycerol; SQDG: sulfoquinovosyldiacylglycerol; PG: diacylglycererylphosphoglycerol. Values are the mean ± standard deviation of three independent cultures (n=3). Blue bars, low light; red bars, high light.
